# Population genetic structure analysis and forensic evaluation of Xinjiang Uigur ethnic group on genomic deletion and insertion polymorphisms

**DOI:** 10.1186/s40064-016-2730-3

**Published:** 2016-07-15

**Authors:** Ting Mei, Chun-Mei Shen, Yao-Shun Liu, Hao-Tian Meng, Yu-Dang Zhang, Yu-Xin Guo, Qian Dong, Xin-Xin Wang, Jiang-Wei Yan, Bo-Feng Zhu, Li-Ping Zhang

**Affiliations:** Department of Biochemistry and Molecular Biology, Basic Medicine College, Xinjiang Medical University, Urumqi, 830011 People’s Republic of China; Key Laboratory of Shaanxi Province for Craniofacial Precision Medicine Research, College of Stomatology, Xi’an Jiaotong University, Xi’an, Shaanxi 710004 People’s Republic of China; Clinical Reaserch Center of Shaanxi Province for Dental and Maxillofacial Diseases, College of Stomatology, Xi’an Jiaotong University, Xi’an, Shaanxi 710004 People’s Republic of China; Institute of Brain and Behavioral Sciences, College of Life Sciences, Shaanxi Normal University, Xi’an, Shaanxi 710062 China; Blood Center of Shaanxi Province, Xi’an, 710061 People’s Republic of China; Key Laboratory of Genome Sciences, Beijing Institute of Genomics, Chinese Academy of Sciences, Beijing, 100101 People’s Republic of China

**Keywords:** Forensic genetics, Population structure, Uigur ethnic group, Genetic relationship

## Abstract

**Background:**

The Uigur ethnic minority is the largest ethnic group in the Xinjiang Uygur Autonomous Region of China, and valuable resource for the study of ethnogeny. The objective of this study was to estimate the genetic diversities and forensic parameters of 30 insertion-deletion loci in Uigur ethnic group from Xinjiang Uigur Autonomous Region of China and to analyze the genetic relationships between Xinjiang Uigur group and other previously published groups based on population data of these loci.

**Results:**

All the tested loci were conformed to Hardy–Weinberg equilibrium after Bonferroni correction. The observed and expected heterozygosity ranged from 0.3750 to 0.5515; and 0.4057 to 0.5037, respectively. The combined power of discrimination and probability of exclusion in the group were 0.99999999999940 and 0.9963, respectively. We analyzed the *D*_*A*_ distance, interpopulation differentiations and population structure, conducted principal component analysis and neighbor-joining tree based on our studied group and 21 reference groups. The present results indicated that the studied Xinjiang Uigur group (represented our samples from the whole territory of Xinjiang Uigur Autonomous Region) had a close relationships with Urumchi Uigur (represented previously reported samples from Urumchi of Xinjiang) and Kazak groups.

**Conclusions:**

The present study may provide novel biological information for the study of population genetics, and can also increase our understanding of the genetic relationships between Xinjiang Uigur group and other groups.

**Electronic supplementary material:**

The online version of this article (doi:10.1186/s40064-016-2730-3) contains supplementary material, which is available to authorized users.

## Background

The short tandem repeats (STRs) are commonly used genetic makers in the field of forensic sciences, and single nucleotide polymorphisms (SNPs) are considered as alternative and supplementary markers to STRs (Gill 2001; Kidd et al. 2005; Tan et al. 2015; Ye et al. 2014). SNPs can be captured in smaller amplicons than STRs without stutter in the profile. Insertion-deletion polymorphisms (InDels) as biallelic polymorphic markers are considered to have potential values in forensic application due to number of advantages properties shared with the similar binary variation of SNPs, for example, smaller amplicons, lower mutation rates than STRs and widely distribute in the human genome (Phillips et al. 2007; Fondevila et al. 2012; Shi et al. 2015; Romanini et al. 2012). At present, InDels have been applied in forensic genetic applications including individual identification (Pereira et al. 2009), inferring biogeographic ancestry (Yang et al. 2005) and population genetic studies et al. (Zaumsegel et al. 2013).

The Investigator DIPplex^®^ kit (Qiagen, Hilden, Germany) contains the following components for the simultaneous amplification of Amelogenin and 30 autosomal InDels (the genomic information regarding chromosomal localization of the 30 InDel loci was shown in Table 1). The allele length variations of the InDels range from 4 to 22 bp and all amplicons are shorter than 160 bp, which make them more suitable for highly degraded DNA samples in forensic caseworks. To date, several populations’ genetic data have been published based on this kit, e.g. Japanese, Poland and Korean groups, and so on (Nunotani et al. 2015; Pepinski et al. 2013; Kim et al. 2014).

**Fig. 1:**
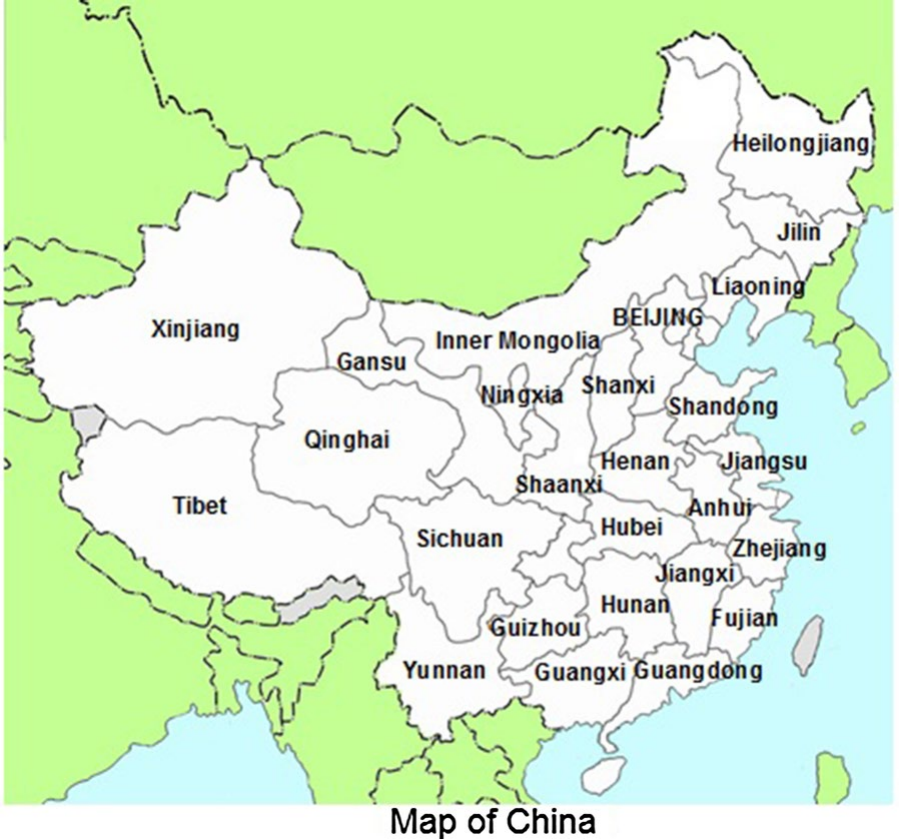
A map showing the geographic location of the Xinjiang Uigur Autonomous Region, China

Xinjiang Uigur Autonomous Region is located in the northwest border of China with the land of 1.6649 million square kilometers and account for one-sixth of China’s total area (Fig. 1). It lies in the heart of the ancient Silk Road which has historically experienced migration of many groups of Eastern and Western Eurasians. The Uigur, as the main nationality of Xinjiang Uigur Autonomous Region, has a population of 10.06 million in 2010 (http://www.stats.gov.cn/tjsj/pcsj/rkpc/6rp/indexch.htm). The Uigurs mainly live in Kashi which is located in the south of Tianshan Mountain, and others are scattered in Ili and Urumchi area. Uigurs have their own language and words and their language belongs to the Turkic branch of Altaic language family. The belief of the Uigurs is Islamism which has a great influence on Uigurs’ culture and custom (Shan and Deng 2012). In the present study, we obtained the population genetic data and calculated the forensic parameters of 30 InDels in the studied Xinjiang Uigur group. We also collected the population data from previously reported groups to analysis their genetic relationships including Uigurs living in different area, other groups in China, Asian, European and Amerindian groups.

## Methods

### Sample collection and DNA extraction

A total of 136 bloodstain samples were collected from Xinjiang Uigur Autonomous Region. All volunteers resided in Xinjiang Uigur Autonomous Region for more than three generations and signed the informed consents before being involved in the study. This study was approved by Institutional Ethics Committee, Xinjiang Medical University, China. Genomic DNA was extracted from bloodstained samples using the Chelex-100 method according to Walsh et al. (1991).

### Amplification and genotyping

Amplification of 30 InDel loci was performed using the Investigator DIPplex^®^ kit on GeneAmp PCR System 9700 Thermal Cycler (Applied Biosystems, Foster City, CA, USA) according to the Investigator DIPplex handbook instruction. Amplification products were separated via capillary electrophoresis on an ABI3500 Genetic Analyzer (Applied Biosystems, Foster City, CA, USA) according to manufacturer’s instruction. The control DNA 9948 (Promega, Madison, WI, USA) was analyzed as positive control. Genotyping results were obtained using the software GeneMapper v3.2 (Applied Biosystems, Foster City, CA, USA) by comparing to allelic ladder.

### Reference groups

InDel data from 21 previously published groups including 9 Chinese groups: Beijing Han, Tibetan, Kazak, Urumchi Uigur (represented previously reported samples from Urumchi of Xinjiang) (Wei et al. 2014), Guangdong Han (Hong et al. 2013), Shanghai Han, She (Wang et al. 2014), Yi (Zhang et al. 2015) and Xibe (Meng et al. 2015); 6 Mexican groups: Chihuahua Mexican, Mexico Mexica, Jalisco Mexican, Veracruz Mexican, Yucatan Mexican and Mexican Amerindian (Martínez-Cortés et al. 2015); South Korean (Korea) (Seong et al. 2014); Dane (Denmark) (Friis et al. 2012); Two Spanish groups: Basque and Central Spanish (Martín et al. 2013); Uruguayan (Uruguay) (Saiz et al. 2014); and Hungarian (Hungary) (Kis et al. 2012) were collected for population genetic analysis.

### Quality control

We strictly followed International Society for Forensic Genetics (ISFG) recommendations on the analysis of the DNA polymorphisms (Schneider 2007).

### Statistical analysis

Allele frequencies and forensic parameters including observed heterozygosity (Ho), Hardy–Weinberg equilibrium (HWE), match probability (MP), polymorphic information content (PIC), power of exclusion (PE), discrimination power (PD) and typical paternity index (TPI) were estimated by the modified Powerstat v1.2 spreadsheet (Promega, Madison, WI, USA). Expected heterozygosity (He) was calculated according to the formula: $He = \frac{n}{n - 1}\left( {1 - \sum\nolimits_{i = 1}^{k} {p_{i}^{2} } } \right)$ (Nei 1978), *p*_*i*_ was the allele frequency of allele *i, k* was the number of alleles and *n* was the number of samples. The pairwise *Fst* and *p* values were calculated by Arlequin statistical software v.3.5 (Excoffier and Lischer 2010). Principal component analysis (PCA) based on allele frequencies was evaluated in MATLAB2007a (MathWorks Inc., USA). Linkage disequilibrium (LD) analysis was performed using the SNP Analyzer V2.0 (Istech, South Korea) (Yoo et al. 2008). The *D*_*A*_ distances were obtained using the DISPAN program (Ota 1993). According to the *D*_*A*_ distances the neighbor-joining (NJ) tree was conducted. Population structure analysis was conducted by the STRUCTURE program (version 2.2) using Admixture Model with parameters adjusted to: burn-in-period, 100,000; run time, 100,000 steps in the Markov Chain; K values, 2–7; and iteration time, 15 (Pritchard et al. 2003; Jakobsson and Rosenberg 2007).

## Results and discussion

### Forensic parameter analysis

All studied loci were found to be in accordance with HWE in Xinjiang Uigur group after Bonferroni correction when the significance level was adjusted to 0.0017 (*p* = 0.05/30). The allele frequencies and forensic parameters of 30 InDel loci in Xinjiang Uigur group were shown in Table 1; and the raw genotyping data were shown in Additional file 1: Table S1. The Ho and He ranged from 0.3750 (HLD56 and HLD84) to 0.5515 (HLD83, HLD92 and HLD131); 0.4057 (HLD64) to 0.5037 (HLD101), respectively. The PIC, TPI, PD and PE values ranged from 0.3216 to 0.3750; 0.8000 to 1.1148; 0.5563 to 0.6513 and 0.0994 to 0.2366, respectively. The highest and lowest MP were 0.4437 (HLD64) and 0.3487 (HLD125), respectively. The combined power of discrimination (CPD) and probability of exclusion (CPE) in the group were 0.99999999999940 and 0.9963, respectively. The high CPD value demonstrates that the panel of 30 InDel loci had potential in forensic individual identification.

**Table 1:** Allele frequencies and forensic parameters for 30 InDels in Uigur group from Xinjiang Uigur Autonomous Region (n = 136)

HLD	rs#	Chromosome localization	DIP+	DIP−	MP	PD	PIC	PE	TPI	Ho	He	HWE
6	1610905	16q13	0.5184	0.4816	0.3539	0.6461	0.3747	0.1463	0.9067	0.4485	0.5030	0.2196
39	17878444	1p22.1	0.2978	0.7022	0.4303	0.5697	0.3308	0.1359	0.8831	0.4338	0.4213	0.7398
40	2307956	1p32.3	0.5515	0.4485	0.3803	0.6197	0.3723	0.1875	1.0000	0.5000	0.4984	0.9354
45	2307959	2q31.1	0.6066	0.3934	0.3874	0.6126	0.3634	0.1689	0.9577	0.4779	0.4808	0.9798
48	28369942	2q11.2	0.4007	0.5993	0.3729	0.6271	0.3650	0.1463	0.9067	0.4485	0.4839	0.4338
56	2308292	4q25	0.6581	0.3419	0.3859	0.6141	0.3488	0.0994	0.8000	0.3750	0.4533	0.0724
58	1610937	5q14.1	0.4154	0.5846	0.3729	0.6271	0.3677	0.1573	0.9315	0.4632	0.4893	0.5714
64	1610935	5q12.3	0.7206	0.2794	0.4437	0.5563	0.3216	0.1308	0.8718	0.4265	0.4057	0.5961
67	1305056	5q33.2	0.6140	0.3860	0.4047	0.5953	0.3617	0.1940	1.0149	0.5074	0.4775	0.4608
70	2307652	6q16.1	0.5699	0.4301	0.3965	0.6035	0.3701	0.2075	1.0462	0.5221	0.4939	0.4839
77	1611048	7q31.1	0.4890	0.5110	0.3716	0.6284	0.3749	0.1811	0.9855	0.4926	0.5035	0.8346
81	17879936	7q21.3	0.6471	0.3529	0.3991	0.6009	0.3524	0.1517	0.9189	0.4559	0.4601	0.9524
83	2308072	8p22	0.3640	0.6360	0.4417	0.5583	0.3558	0.2366	1.1148	0.5515	0.4664	0.0425
84	3081400	8q24.12	0.6213	0.3787	0.3654	0.6346	0.3598	0.0994	0.8000	0.3750	0.4740	0.0230
88	8190570	9q22.32	0.4632	0.5368	0.3854	0.6146	0.3736	0.2007	1.0303	0.5147	0.5010	0.7164
92	17174476	11q22.2	0.4816	0.5184	0.4054	0.5946	0.3747	0.2366	1.1148	0.5515	0.5030	0.2407
93	2307570	12q22	0.5257	0.4743	0.3881	0.6119	0.3743	0.2075	1.0462	0.5221	0.5024	0.6153
97	17238892	13q12.3	0.3382	0.6618	0.3949	0.6051	0.3475	0.1212	0.8500	0.4118	0.4510	0.3787
99	2308163	14q23.1	0.6801	0.3199	0.4181	0.5819	0.3404	0.1463	0.9067	0.4485	0.4383	0.7809
101	2307433	15q26.1	0.5000	0.5000	0.3616	0.6384	0.3750	0.1630	0.9444	0.4706	0.5037	0.4660
111	1305047	17p11.2	0.3346	0.6654	0.4080	0.5920	0.3461	0.1463	0.9067	0.4485	0.4486	0.9695
114	2307581	17p13.3	0.3199	0.6801	0.4134	0.5866	0.3404	0.1359	0.8831	0.4338	0.4383	0.9461
118	16438	20p11.1	0.6066	0.3934	0.3941	0.6059	0.3634	0.1811	0.9855	0.4926	0.4808	0.7505
122	8178524	21q22.11	0.4485	0.5515	0.3803	0.6197	0.3723	0.1875	1.0000	0.5000	0.4984	0.9354
124	6481	22q12.3	0.6360	0.3640	0.3902	0.6098	0.3558	0.1463	0.9067	0.4485	0.4664	0.7053
125	16388	22q11.23	0.4890	0.5110	0.3487	0.6513	0.3749	0.1359	0.8831	0.4338	0.5035	0.1139
128	2307924	1q31.3	0.4338	0.5662	0.4087	0.5913	0.3706	0.2291	1.0968	0.5441	0.4949	0.2336
131	1611001	7q36.2	0.4890	0.5110	0.4050	0.5950	0.3749	0.2366	1.1148	0.5515	0.5035	0.2448
133	2067235	3p22.1	0.4449	0.5551	0.3848	0.6152	0.3719	0.1940	1.0149	0.5074	0.4976	0.7865
136	16363	22q13.1	0.4779	0.5221	0.3689	0.6311	0.3745	0.1750	0.9714	0.4853	0.5027	0.7164

*HLD* human locus deletion/insertion polymorphism, *DIP*− frequency of short allele, *DIP*+ frequency of long allele, *Ho* observed heterozygosity, *He* expected heterozygosity, *MP* matching probability, *PD* power of discrimination, *PE* probability of exclusion, *PIC* Polymorphic information contents, *TPI* typical paternity index, *HWE* probability value of the exact test for Hardy–Weinberg equilibrium, *p* the short arm of a chromosome, *q* the long arm of a chromosome

### Linkage disequilibrium analysis

Linkage disequilibrium has been tested for all possible combinations between each locus. The linkage disequilibrium pattern revealed by *r*^2^ values between each locus was shown in Additional file 2: Table S2, The results showed that there was no linkage disequilibrium observed among all the loci with the values of *r*^2^ less than 0.1, which indicated those genetic markers were relatively independent for subsequent comparison among 22 groups.

### Clustering analysis

**Fig. 2:**
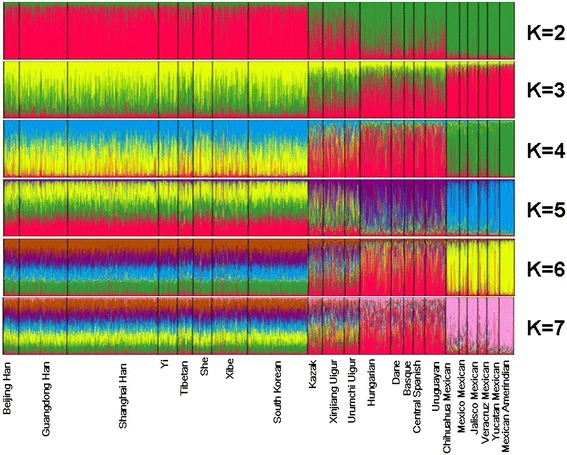
The cluster analysis of 22 groups using the STRUCTURE program based on the genotyping data of 30 InDel loci

Before conducting the comparison, we had re-read the references and made sure that loci in all reference populations showed no deviation from HWE and linkage equilibrium. We analyzed the population structures of Xinjiang Uigur group (represented our samples from the whole territory of Xinjiang Uigur Autonomous Region) and 21 referenced groups and the results were shown in Fig. 2. The Asian groups were separated from both Amerindian groups and European groups at K = 2, the 5 European groups and 6 Amerindian groups constituted almost entirely by green component while 8 Asian groups by red; The Kazak, Urumchi Uigur and Xinjiang Uigur groups displayed admixture constitution of both green and red components. At K = 4, we could clearly separate Amerindian groups from European groups. Uigurs and Kazaks were much better separated from both Europeans and Asians by K = 6.

### Principal component analysis

**Fig. 3:**
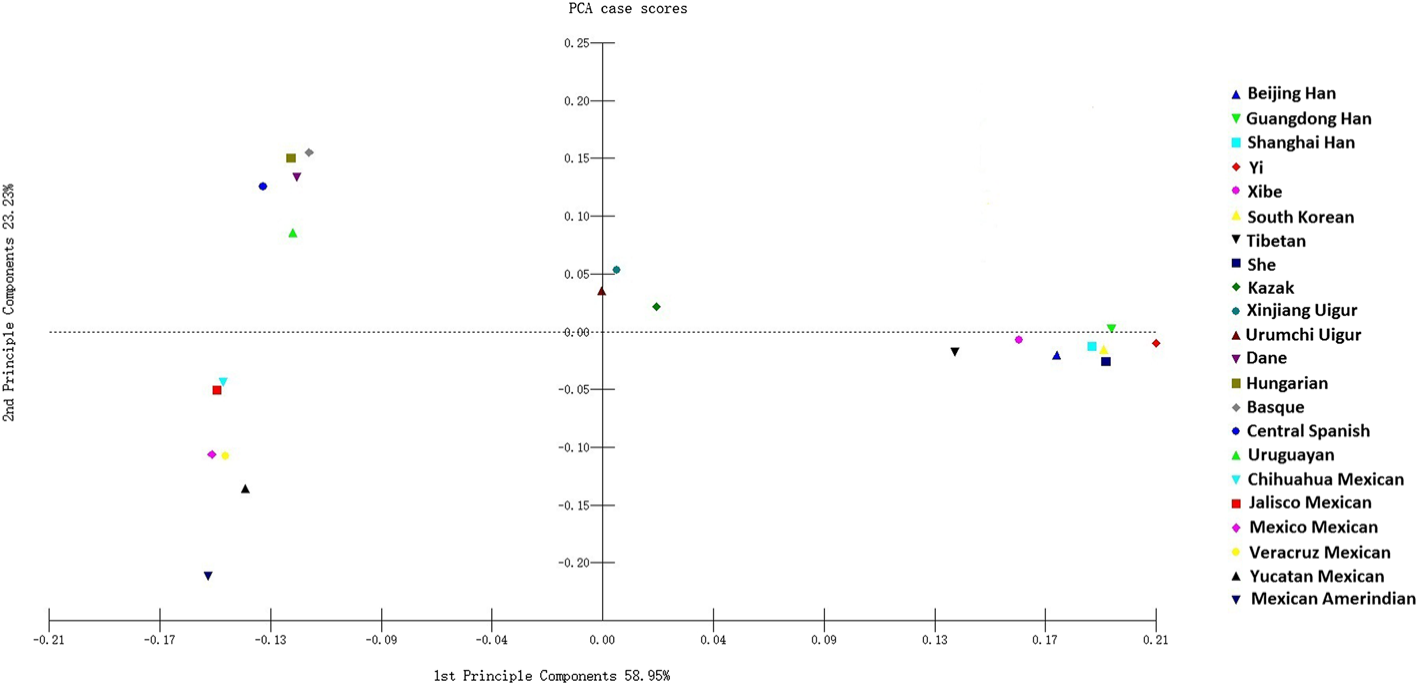
PCA based on population data of 30 InDel loci of Xinjiang Uigur group and 21 reference groups

A PCA was constructed to analyze the relationships between the Xinjiang Uigur group and other 21 groups. The result was shown in Fig. 3. The first and second component accounted for 58.95 and 23.23 %, respectively; and the cumulative contribution of the first two principal components defined 82.18 % of the total variance. In the plot figure, 5 European groups and 6 Amerindian groups located in the left part, while the 8 Asian groups located in the right part and the 3 Eurasian groups (Kazak, Urumchi Uigur and Xinjiang Uigur groups) in the central part. The Xinjiang Uigur group had short distance with the Urumchi Uigur and Kazak groups in PCA plot, which indicated the Xinjiang Uigur group had closer genetic relationships with those two groups.

### Interpopulation differentiations

We estimated pairwise *Fst* and *p*-values utilizing analysis of molecular variance method between Uigur group and previously published groups at the 30 InDel loci, which were given in Additional file 3: Table S3. The results showed that the least differences were found between the Xinjiang Uigur group and the Urumchi Uigur and Kazak groups, with significant differences at one and three loci, respectively; whereas differences were observed between Xinjiang Uigur group and other groups at 5–20 loci. The results indicated that the distribution of allele frequencies in different groups were different. Therefore, InDel would be a useful tool to study the migration patterns, geneflow, admixture and ancestry with the discovery of more available loci (Hefke et al. 2015).

### *D*_*A*_ distance

The *D*_*A*_ distance was calculated to elucidate the genetic distance. The *D*_*A*_ distances between Xinjiang Uigur group and other reference groups were shown in Table 2. According to the *D*_*A*_ distances, the Xinjiang Uigur group was closest to the Urumchi Uigur group (*D*_*A*_ = 0.0012), and followed by the Kazak (*D*_*A*_ = 0.0019) group, both of them belongs to Altaic language family. The greatest distance was detected when comparing the Xinjiang Uigur group with Yucatan Mexican (*D*_*A*_ = 0.0353) and Mexican Amerindian (*D*_*A*_ = 0.0473) groups.

**Table 2:** The *D*_*A*_ distances among the 22 groups based on 30 InDel loci

Populations	Beijing Han	Guangdong Han	Shanghai Han	Yi	Xibe	South Korean	Tibetan	She	Kazak	Urumchi Uigur	Dane	Hungarian	Basque	Central Spanish	Uruguayan	Chihuahua Mexican	Jalisco Mexican	Mexico Mexican	Veracruz Mexican	Yucatan Mexican	Mexican Amerindian
Guangdong Han	0.0019																				
Shanghai Han	0.0011	0.0006																			
Yi	0.0054	0.0038	0.0040																		
Xibe	0.0022	0.0023	0.0015	0.0052																	
South Korean	0.0024	0.0017	0.0008	0.0042	0.0016																
Tibetan	0.0029	0.0055	0.0038	0.0066	0.0037	0.0038															
She	0.0023	0.0015	0.0019	0.0051	0.0032	0.0028	0.0065														
Kazak	0.0083	0.0100	0.0096	0.0133	0.0068	0.0115	0.0074	0.0112													
Urumchi Uigur	0.0100	0.0118	0.0114	0.0163	0.0092	0.0135	0.0093	0.0133	0.0013												
Dane	0.0251	0.0265	0.0264	0.0315	0.0227	0.0288	0.0226	0.0275	0.0093	0.0083											
Hungarian	0.0255	0.0275	0.0271	0.0325	0.0231	0.0295	0.0222	0.0289	0.0084	0.0068	0.0026										
Basque	0.0270	0.0268	0.0270	0.0328	0.0236	0.0287	0.0258	0.0288	0.0111	0.0096	0.0048	0.0045									
Central Spanish	0.0262	0.0269	0.0268	0.0323	0.0226	0.0288	0.0231	0.0285	0.0085	0.0069	0.0030	0.0022	0.0033								
Uruguayan	0.0230	0.0244	0.0240	0.0286	0.0203	0.0258	0.0199	0.0255	0.0067	0.0057	0.0039	0.0021	0.0043	0.0023							
Chihuahua Mexican	0.0445	0.0471	0.0465	0.0522	0.0441	0.0500	0.0422	0.0525	0.0278	0.0241	0.0183	0.0156	0.0212	0.0200	0.0207						
Jalisco Mexican	0.0437	0.0450	0.0448	0.0512	0.0420	0.0484	0.0417	0.0502	0.0260	0.0225	0.0170	0.0137	0.0197	0.0181	0.0189	0.0017					
Mexico Mexican	0.0534	0.0548	0.0544	0.0602	0.0517	0.0576	0.0502	0.0606	0.0345	0.0313	0.0222	0.0210	0.0256	0.0258	0.0274	0.0048	0.0041				
Veracruz Mexican	0.0498	0.0500	0.0502	0.0566	0.0473	0.0535	0.0468	0.0555	0.0301	0.0263	0.0192	0.0160	0.0209	0.0197	0.0223	0.0040	0.0024	0.0030			
Yucatan Mexican	0.0588	0.0599	0.0598	0.0675	0.0569	0.0633	0.0567	0.0655	0.0391	0.0351	0.0267	0.0230	0.0277	0.0279	0.0300	0.0048	0.0046	0.0041	0.0031		
Mexican Amerindian	0.0718	0.0724	0.0732	0.0798	0.0698	0.0770	0.0698	0.0796	0.0519	0.0477	0.0365	0.0317	0.0389	0.0393	0.0427	0.0093	0.0090	0.0087	0.0064	0.0053	
Xinjiang Uigur	0.0101	0.0115	0.0108	0.0156	0.0088	0.0125	0.0086	0.0127	0.0019	0.0012	0.0073	0.0055	0.0090	0.0062	0.0051	0.0246	0.0226	0.0310	0.0262	0.0353	0.0473

Xinjiang Uigur represented our samples from the whole territory of Xinjiang Uigur Autonomous Region. Urumchi Uigur represented previously published samples from Urumchi of Xinjiang

### Phylogenetic analysis

**Fig. 4:**
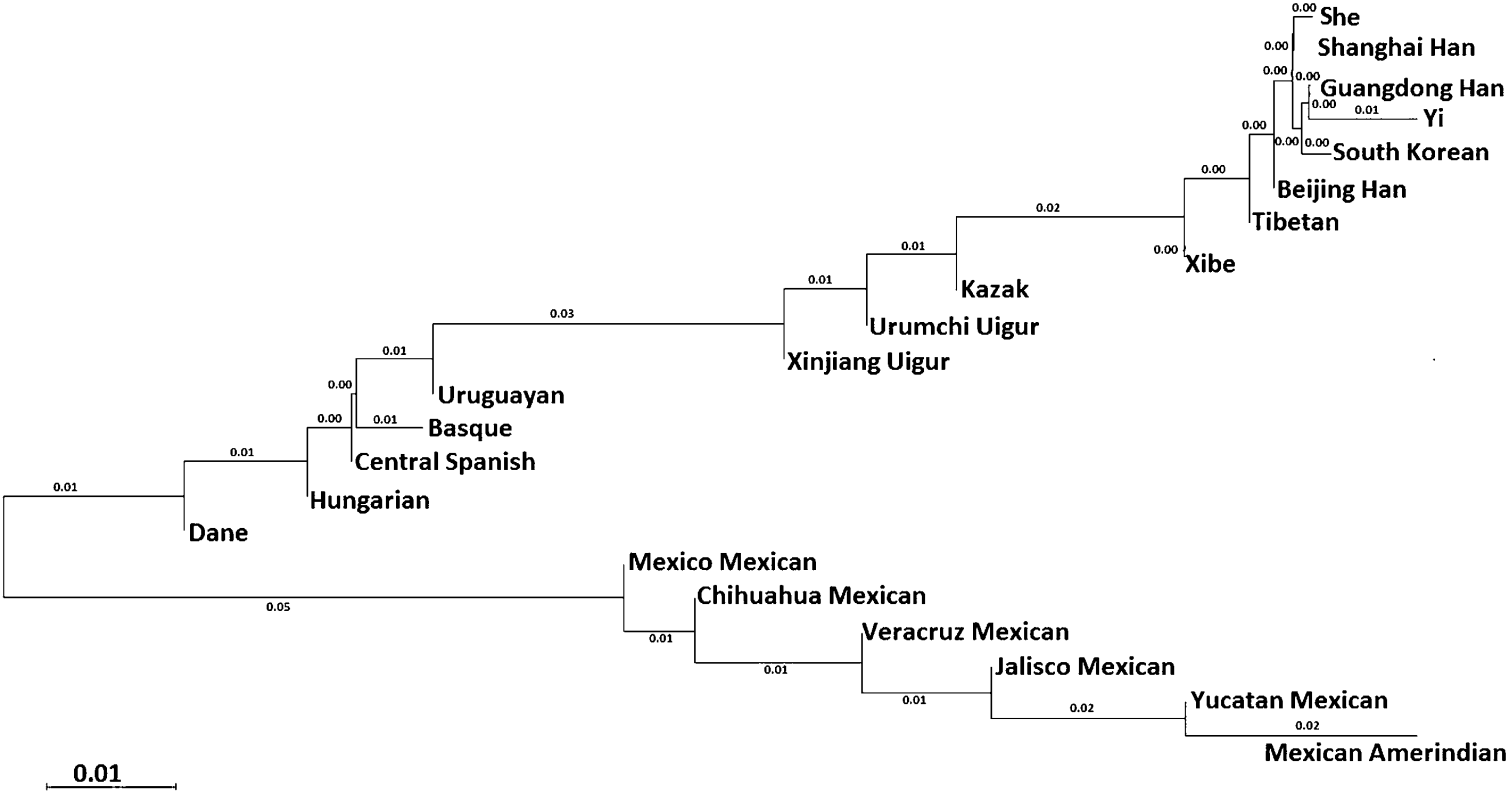
The neighbor-joining tree based on population data of 30 InDel loci of Xinjiang Uigur group and 21 referenced groups

A NJ-tree was constructed based on *D*_*A*_ distances as presented in Fig. 4, the NJ tree showed that the Xinjiang Uigur group was first clustered with the Urumchi Uigur and Kazak groups. The result was consistent with the above mentioned results of STRUCTURE, *D*_*A*_ distance and PCA. According to the relevant historical records, Uigurs were the descendants of ancient Uighur and with large proportion of the descent from Caucasian. Uigurs and Kazaks have common religious belief which indicated that they were likely having the same or similar origin in the process of the formation and development (Palstra et al. 2015; Xu et al. 2006). Therefore, the genetic distances could be relatively close among them. Yuan et al. (2015) studied the genetic polymorphism of 38 STR loci in Uigur group from Southern Xinjiang of China; their *Fst* distance results (21 loci) indicated the Uigur group was closest to Kazak, and our result was similar to theirs.

## Conclusions

In summary, the 30 InDel loci showed relatively high forensic-efficacy in the Xinjiang Uigur group and could be used in forensic individual identification, and also be used as complement for STR loci in forensic paternity testing. The result of *D*_*A*_ distance, STRUCTURE, PCA and NJ tree indicated that the studied Xinjiang Uigur group had a close relationship with Urumchi Uigur and Kazak groups. This study provided valuable data for analysis of genetic relationship and forensic application.

## Additional files

10.1186/s40064-016-2730-3 The genotyping results of the 30 InDel loci from Uigur ethnic group living in Xinjiang Uigur Autonomous Region, China (n = 136).
10.1186/s40064-016-2730-3 The linkage disequilibrium pattern revealed by *r* ^2^ values at 30 InDel loci.c.
10.1186/s40064-016-2730-3 Pairwise *Fst* and *p*-values between Xinjiang Uigur group and other groups at 30 InDel loci (n = 136).
